# Effect of the probiotic strain *Bifidobacterium animalis* subsp. *lactis*, BB-12^®^, on defecation frequency in healthy subjects with low defecation frequency and abdominal discomfort: a randomised, double-blind, placebo-controlled, parallel-group trial

**DOI:** 10.1017/S0007114515003347

**Published:** 2015-09-18

**Authors:** Dorte Eskesen, Lillian Jespersen, Birgit Michelsen, Peter J. Whorwell, Stefan Müller-Lissner, Cathrine M. Morberg

**Affiliations:** 1 Chr. Hansen A/S, 2970 Hørsholm, Denmark; 2 Centre for Gastrointestinal Sciences, University Hospital of South Manchester, Manchester M23 9LT, UK; 3 Park-Klinik Weissensee, 13086 Berlin, Germany

**Keywords:** *Bifidobacterium animalis* subsp. *lactis*, Probiotics, Defecation frequency, Bowel habits, Gastrointestinal well-being

## Abstract

The aim of the present study was to investigate the effect of *Bifidobacterium animalis* subsp. *lactis*, BB-12^®^, on two primary end points – defecation frequency and gastrointestinal (GI) well-being – in healthy adults with low defecation frequency and abdominal discomfort. A total of 1248 subjects were included in a randomised, double-blind, placebo-controlled trial. After a 2-week run-in period, subjects were randomised to 1 or 10 billion colony-forming units/d of the probiotic strain BB-12^®^ or a matching placebo capsule once daily for 4 weeks. Subjects completed a diary on bowel habits, relief of abdominal discomfort and symptoms. GI well-being, defined as global relief of abdominal discomfort, did not show significant differences. The OR for having a defecation frequency above baseline for ≥50 % of the time was 1·31 (95 % CI 0·98, 1·75), *P*=0·071, for probiotic treatment overall. Tightening the criteria for being a responder to an increase of ≥1 d/week for ≥50 % of the time resulted in an OR of 1·55 (95 % CI 1·22, 1·96), *P*=0·0003, for treatment overall. A treatment effect on average defecation frequency was found (*P*=0·0065), with the frequency being significantly higher compared with placebo at all weeks for probiotic treatment overall (all *P*<0·05). Effects on defecation frequency were similar for the two doses tested, suggesting that a ceiling effect was reached with the one billion dose. Overall, 4 weeks’ supplementation with the probiotic strain BB-12^®^ resulted in a clinically relevant benefit on defecation frequency. The results suggest that consumption of BB-12^®^ improves the GI health of individuals whose symptoms are not sufficiently severe to consult a doctor (ISRCTN18128385).

In recent years, the gastrointestinal (GI) microbiota has been suggested to be implicated in the pathophysiology of multifactorial functional bowel disorders such as irritable bowel syndrome (IBS) and constipation^(^ ^1^ ^–^ ^3^ ^)^, and as a consequence probiotics have been suggested as a potential means to manage symptoms of IBS and maintain healthy bowel habits^(^ ^4^ ^,^ ^5^ ^)^. Patients with these disorders may present with altered bowel habits such as low defecation frequency, hard stools and incomplete defecation or with symptoms such as abdominal pain, discomfort and bloating^(^ ^6^ ^,^ ^7^ ^)^. The conditions are often undiagnosed and self-managed by the patient^(^ ^8^ ^,^ ^9^ ^)^ and pose a heavy burden on the individual and society^(^ ^10^ ^–^ ^12^ ^)^. It can be a challenge to distinguish patients with IBS from healthy people with similar GI symptoms, as healthy people may have the same complaints, only less frequently and less severe^(^ ^8^ ^,^ ^13^ ^,^ ^14^ ^)^. Conducting trials in IBS patients or healthy populations with GI complaints is difficult because no biomarkers exist and they rely solely on patient report of symptoms. It is currently not clear which outcomes are most valid to use, and as the available tools are not ideal new tools are under development^(^ ^15^ ^,^ ^16^ ^)^. Clinical trials are further complicated by a potentially very high placebo response rate^(^ ^17^ ^)^, and studying healthy subjects with mild GI symptoms entails the additional difficulty of measuring improvements of already low symptom scores or a suboptimal defecation frequency within the normal range. No guidelines exist for clinical trials in healthy populations with GI complaints; however, due to the similarities with IBS, it is relevant to apply guidelines for IBS trials when designing studies in healthy individuals^(^ ^7^ ^,^ ^17^ ^,^ ^18^ ^)^.

The potential of probiotics to improve bowel habits in healthy populations with low defecation frequency has been examined in a limited number of studies. The effect of the probiotic strain *Bifidobacterium animalis* subsp. *lactis*, BB-12^®^, on defecation frequency has been examined in young adults and in the elderly, demonstrating significant improvements compared with placebo treatment^(^ ^19^ ^–^ ^21^ ^)^. In IBS trials, subjects’ assessment of global relief has until recently been the generally accepted primary outcome variable^(^ ^7^ ^,^ ^17^ ^,^ ^18^ ^)^ and has been investigated in several trials in IBS patients^(^ ^1^ ^,^ ^22^ ^–^ ^24^ ^)^ and in probiotic studies in healthy subjects with minor GI complaints^(^ ^25^ ^,^ ^26^ ^)^. We, therefore, set out with the primary objective to investigate the effect of the probiotic strain BB-12^®^ in different dosages on defecation frequency and global relief in healthy subjects with low defecation frequency and abdominal discomfort. The secondary objective was to investigate the effect on abdominal pain and bloating. As little is known about healthy populations with minor GI complaints, we also wanted to explore whether certain subgroups were more likely to benefit from treatment with the probiotic strain BB-12^®^.

## Methods

### Study design

The study was a randomised, double-blind, placebo-controlled, parallel-group study performed in eight centres in France, Germany and the UK between September 2010 and December 2012.

The study comprised a 2-week run-in period and a 4-week intervention period. During each of the two periods, the subjects completed a diary on a daily basis for the assessment of study outcomes and compliance with the study treatment.

### Ethics and study population

The study was performed in accordance with the principles of the Declaration of Helsinki and Good Clinical Practice. All the procedures involving human subjects were approved by the relevant Ethics Committees for each site and in France also from the French Agency for the Safety of Health Products (Agence française de sécurité sanitaire des produits de santé, AFSSAPS). Written informed consent was obtained from all the subjects. The study is registered on the ISRCTN database (International Standard Registered Clinical/social study number, www.isrctn.com) (ISRCTN18128385).

Subjects were recruited from volunteer databases and by advertisements and flyers. Eligible subjects were healthy men and women, 18–70 years old, with a low defecation frequency (2–4 d/week) and complaints of general abdominal discomfort. Most important exclusion criteria were history or diagnosis of GI disease, IBS or complicated GI surgery, depressive disorder, use of oral antibiotics within 4 weeks before the screening visit and the use of drugs, large doses of vitamins and minerals or food or herbal supplements for digestive symptoms, unless in stable dose. A complete list of inclusion and exclusion criteria is provided in the online Supplementary Table S1.

Subjects were randomised if they had an average defecation frequency of 2–4 d/week during the run-in period and a weekly average composite GI symptom score of a minimum of 5 or 5 d where a minimum of one symptom was of at least severe intensity.

### Study products

*Bifidobacterium animalis* subsp. *lactis*, BB-12^®^ (DSM15954), was provided in capsules with 1 or 10 billion colony-forming units/capsule. Placebo products were identical capsules without any probiotics. The active and placebo products had similar appearance, taste and smell and were provided in identical containers with identical labelling. Subjects took one capsule once daily with their breakfast. Study products were produced at Chr. Hansen A/S.

### Restrictions during the study

Concomitant medication was allowed as long as the dose remained stable from screening to the end of the study. During the entire study period, subjects were not allowed to consume any probiotics or fermented dairy products and were told to avoid excessive physical exercise and drastic changes in diet or lifestyle.

### Randomisation

The randomisation list was generated by a statistician not involved in the study using the computer programmes RANCODE version 3.6 (IDV) and SAS version 8.2 (SAS Institute Inc.). Randomisation to the three groups was performed in a 1:1:1 ratio in blocks of six and stratified by sex and hormonal status, resulting in three strata (men, pre-menopausal and post-menopausal women). Study products were labelled according to the randomisation lists and only identified by the randomisation number. Subject allocation was performed by the Investigator in consecutive order by assigning eligible subjects the first available randomisation number for the relevant stratum. All the subjects, Investigators, CRO and Sponsor staff involved in the study were blinded until the final database was locked. Only the Independent Data Monitoring Committee (IDMC) and the study supply coordinator at Chr. Hansen A/S had access to the randomisation list to perform interim analyses and labelling of the study products, respectively.

### Data collection

Each day, the subjects completed a Bristol Stool Form in their diary to provide data on stool form^(^ ^7^ ^)^. Data on defecation frequency were obtained by counting the days per week with a completed Bristol Stool Form. As a measure of GI well-being, we assessed subjects’ global relief of general abdominal discomfort^(^ ^7^ ^)^. At the end of each week during the intervention period, subjects answered the following question: ‘How would you consider your general abdominal discomfort in the past 7 d compared to the month before beginning the consumption of study product?’, with the response options ‘markedly relieved’, ‘somewhat relieved’, ‘unchanged’, ‘somewhat worsened’ and ‘markedly worsened’.

Abdominal pain and bloating were rated daily during the entire study period on a Likert scale with the response options 0 (no), 1 (mild), 2 (moderate), 3 (severe) and 4 (unbearable).

Before and after the intervention, subjects completed questionnaires on their physical activity level (International Physical Activity Questionnaire short form)^(^ ^27^ ^)^. All the subjects completed a 3 d food diary during the run-in period and the last week of the intervention period.

All adverse events (AE), defined as any untoward medical occurrence in a study subject during the intervention period, were recorded.

### Statistical methods

#### Sample size

The sample size calculation was based on a one-sided *α* level of 0·0125 to account for two primary end points, an assumed placebo response rate of 40 % and a treatment difference of 10 % in responder rates based on previously published studies^(^ ^23^ ^,^ ^28^ ^)^. Further, accounting for a potential 10 % dropout rate and three interim analyses, 580 subjects in each group were required, totalling to 1740 subjects.

#### Interim analysis

A group sequential design (GSD) was used allowing for early stopping of a dose group or the study for futility or early efficacy. The interim analyses were planned after approximately 60, 74 and 89 % of the subjects had completed the study and were conducted by an IDMC ensuring that all persons engaged in the study were kept blinded. A conservative O’Brien–Fleming approach was used, spending rather little *α* at the interim looks and actual boundary values for each interim look calculated using Proc SEQDESIGN in SAS. The interim analyses were performed within a closed testing hierarchy, where the doses were ranked, testing the high dose first and the low dose second, and using statistical models identical to the final efficacy analysis. Both primary end points should be statistically significant to conclude early efficacy or futility as opposed to the final efficacy analysis where success was achieved if one of the primary end points was significant.

#### Statistical analysis

All the analyses were performed on the intention-to-treat (ITT) population. As supportive analyses, the two primary end points were analysed for the per-protocol (PP) population, excluding subjects who had protocol deviations with potential impact on the efficacy end points.

Owing to the GSD and the stopping rules applied during the interim analyses, the primary efficacy analysis was performed one-sided using a significance level of 2·5 %. All the other statistical tests were assessed using a two-sided significance level of 5 %. For all variables, pairwise comparisons of the two doses of the test product compared with the placebo were performed. SAS version 9.2 for Windows (SAS Institute Inc.) was used for all the analyses.

#### Primary end points

The main analysis for the two primary end points – defecation frequency and GI well-being – was based on responder rates^(^ ^7^ ^,^ ^17^ ^,^ ^18^ ^)^. For defecation frequency, a responder was defined as a subject with an average weekly defecation frequency above baseline for at least 50 % of the time, and for GI well-being a responder was defined as a subject who achieved relief (having answered ‘markedly relieved’ or ‘somewhat relieved’) for at least 50 % of the time – that is, for at least 2 weeks of the 4-week treatment period. All available data from the 28 d intervention period were used, and no imputation of missing data was performed.

As both end points are binary responder end points, identical logistic regression models using Proc GENMOD in SAS were used. The models included a single covariate representing the three different groups of sex/hormonal status. The main analysis for each of the two primary end points was adjusted for the *α*-spending during the interim analyses and for multiplicity using a closed Bonferroni–Holm procedure to ensure an overall one-sided significance level of 2·5 % for the interim looks and final analyses combined^(^ ^29^ ^)^. The output was responder rates and 95 % CI in each dose group, OR and 95 % CI for the chance of being a responder along with *P* values. All *P* values reported in this study are two-sided.

Analyses of the average weekly number of days with defecation and the raw GI well-being scores were predefined as exploratory supportive analyses for the two primary end points. Repeated generalised estimation equations (GEE) models including terms for week, interaction between treatment and week and sex/hormonal status were used; for defecation frequency the baseline value was also included^(^ ^17^ ^,^ ^30^ ^)^.

#### Subgroup analyses of primary end points

Subgroups of different baseline defecation frequency (<3 and ≥3 d/week) and sex/hormonal status (men, pre-menopausal and post-menopausal women) were predefined, and subgroup analyses were performed for responder analysis of the two primary end points by including terms for the subgroup and the interaction between the subgroup and treatment in the statistical models.

#### *Post hoc* analysis of responders in defecation frequency

Based on recently issued IBS guidelines, defining a weekly responder as a patient with an increase from baseline of at least one complete spontaneous bowel movement per week^(^ ^31^ ^,^ ^32^ ^)^, we performed a *post hoc* analysis tightening the criteria for efficacy by defining a weekly responder as a subject with an increase in defecation frequency from baseline of at least 1 d/week for at least 50 % of the time. Finally, as there was no difference in odds ratios or average defecation frequency between the two doses, an overall treatment effect was estimated using statistical models where the active treatment groups were pooled into one. All the *post hoc* analyses were performed in line with the predefined analyses using similar statistical models.

#### Analysis of other end points

The key secondary end points were symptom severity scores for abdominal pain and bloating. For each symptom, a weekly sum was calculated using all available values for the given week with missing values for ≤3 d imputed with the average score of the available days. Analysis of the weekly sum score at week 4 was performed using ANOVA of ranked data with sex/hormonal status and the baseline value as covariates. For stool consistency, the weekly median stool type was calculated using all bowel movements. Statistical analysis of stool consistency at week 4 was performed using ANOVA on ranked data, including sex/hormonal status and the median stool type over the run-in period as covariates.

## Results

### Subject disposition and compliance with study treatment

**Fig. 1:**
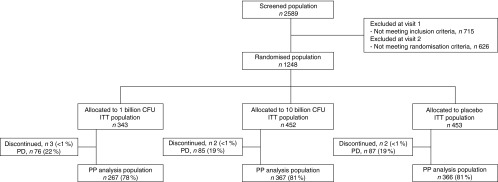
Consort flow chart. CFU, colony-forming units; ITT, intention-to-treat; PD, protocol deviations; PP, per-protocol.

A total of 1248 subjects were randomised into the study and analysed in the ITT analysis. Less than 1 % of the subjects were withdrawn from the study and 1000 subjects (80 %) were included in the PP analysis. As the one billion treatment arm was closed after the first interim analysis and the ten billion treatment arm was closed after the second interim analysis, the number of subjects in the three treatment groups is different (Fig. 1).

The characteristics of the three study groups were similar at baseline (Table 1). During the study, there were no changes in physical activity level or food intake and the use of concomitant medication was similar between the study groups (data not shown).

**Table 1:** Baseline characteristics (intention-to-treat population) (Mean values and standard deviations; numbers and percentages; odds ratios and 95 % confidence intervals; *n* 1248)

	1 billion CFU (*n* 343)		10 billion CFU (*n* 452)		Placebo (*n* 453)	
	Mean	sd	Mean	sd	Mean	sd
Age (years)	37·1	12·7	37·1	12·5	37·4	2·7
BMI (kg/m^2^)	24·4	3·7	24·3	3·6	24·4	3·6
Caucasian (% of *n*)	95·3		98·2		97·1	
Smokers (% of *n*)	26·8		25·9		27·2	
Sex (% of *n*)						
Pre-menopausal women	65·9		63·3		63·8	
Post-menopausal women	15·5		15·7		15·0	
Men	18·7		21·0		21·2	
Physical activity level* (% of *n*)						
Low	23·4		19·8		24·3	
Moderate	47·8		49·2		44·0	
High	28·8		31·0		31·7	
Bowel habits						
Not satisfied with bowel habits (% of *n*)	97·7		97·6		96·7	
Baseline defecation frequency† (d/week)						
OR	2·90		2·91		2·87	
95 % CI	2·8, 3·0		2·8, 3·0		2·8, 2·9	
Baseline defecation frequency <3 d/week (% of *n*)	42·6		43·4		46·1	
Number of complete bowel movements/week†	1·00	1·01	1·04	0·99	1·00	0·99
Severity of abdominal symptoms†						
Pain (weekly sum)						
OR	11·1		11·4		11·0	
95 % CI	10·6, 11·6		10·9, 11·9		10·5, 11·5	
Bloating/distension (weekly sum)						
OR	13·8		14·3		14·2	
95 % CI	13·4, 14·2		13·9, 14·7		13·8, 14·6	
Composite symptom score‡	51·7	16·0	54·7	19·1	53·6	18·7

CFU, colony-forming units.

* Physical activity level based on International Physical Activity Questionnaire scores.

† Baseline values are averages over the 2-week run-in period.

‡ Composite symptom scores included scores on pain, bloating, flatulence, rumbling, nausea and other abdominal discomfort.

Compliance was calculated for the 4-week intervention period based on the number of returned capsules and the subjects’ recordings in the diary of capsules taken. Compliance was >100 % in all the three treatment groups (total mean 102·0 (sd 4·5) %).

### Defecation frequency

**Fig. 2:**
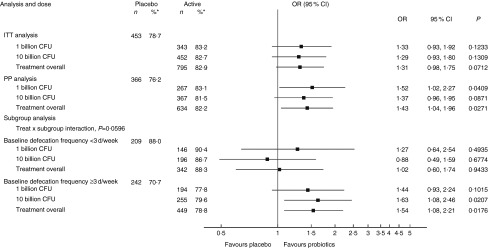
Responders for defecation frequency (intention-to-treat (ITT) and per-protocol (PP) analysis). A responder was defined as a subject with a weekly defecation frequency above baseline for at least 50 % of the time – that is, for at least 2 of the 4-week treatment period; due to missing data, six subjects (0·5 %) could not be classified as responders or non-responders. CFU, colony-forming units; OR, OR for being a responder. * Number of subjects (% responders).

The OR for having a defecation frequency above baseline for at least 2 of the 4-week treatment period was 1·31 (95 % CI 0·98, 1·75) for probiotic treatment overall with similar OR in the one and ten billion dose groups (Fig. 2). In the PP population, comprising 80 % of the population, there was a statistically significant effect in the one billion group and for treatment overall, whereas a similar trend was observed in the ten billion group (Fig. 2). A trend for different OR for being a responder was found in the two subgroups with different defecation frequencies at baseline (*P*=0·060). In the subgroup with a baseline defecation frequency of ≥3 d/week, OR between 1·44 and 1·63 were observed, whereas no significant effect was observed in the subgroup with a baseline defecation frequency <3 d/week (Fig. 2). There was no significant difference between the subgroups of sex/hormonal status (*P*=0·44), and no significant differences to placebo were observed for any subgroup or dose (online Supplementary Fig. S1).

**Fig. 3:**
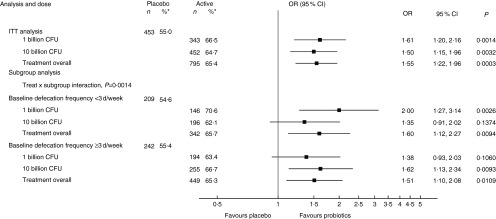
Responders for defecation frequency with tightened responder criteria (intention-to-treat (ITT) analysis). A responder was defined as a subject with a weekly defecation frequency ≥1 d/week above baseline for at least 50 % of the time – that is, for at least 2 of the 4-week treatment period; due to missing data, six subjects (0·5 %) could not be classified as responders or non-responders. CFU, colony-forming units; OR, OR for being a responder. * Number of subjects (% responders).

*Post hoc* responder analysis, defining responders as subjects with an increase in defecation frequency of ≥1 d/week for at least 50 % of the time, showed a statistically significant increase in the probability of being a responder with OR between 1·50 and 1·61 for each dose and treatment overall (Fig. 3). For the two subgroups with different baseline defecation frequency, there was a statistically significant interaction between treatment and subgroup (*P*=0·0014); however, in both subgroups, rather similar and significant OR between 1·51 and 1·60 were observed for treatment overall (Fig. 3). For the subgroups of sex/hormonal status, there was a statistically significant interaction between treatment and subgroup (*P*=0.034), whereas the OR for being a responder were more similar between the subgroups (range 1·40–1·84) than with the initial responder definition (online Supplementary Fig. S1).

**Fig. 4:**
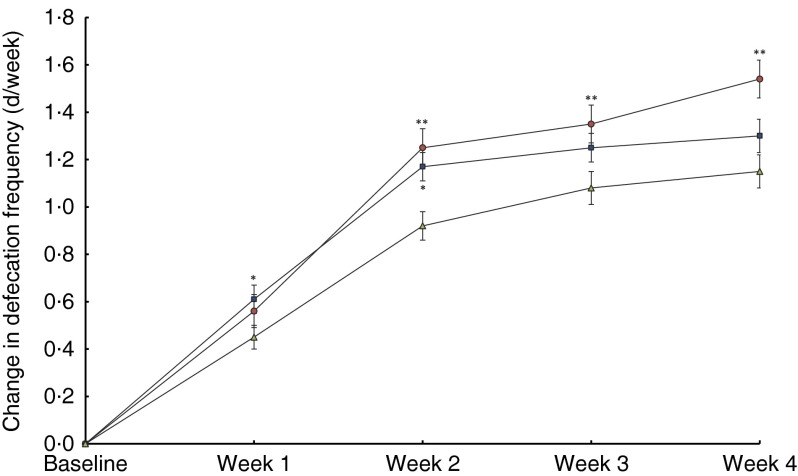
Weekly changes from baseline in defecation frequency (intention-to-treat population). Values are means with their standard errors. Defecation frequency recorded in subject diaries and reported as days per week with defecation. Overall treatment effect (*P*=0·0054). The one billion group is significantly different from the placebo group at weeks 2, 3 and 4 (** *P*<0·01). The ten billion group is significantly different from the placebo group at weeks 1 and 2 (* *P*<0·05). CFU, colony-forming units. 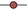, 1 billion CFU, *n* 343; 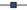, 10 billion CFU, *n* 452; 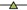, placebo, *n* 453.

The average defecation frequency increased over time in all the groups (*P*<0·0001) from an average of approximately 3 d/week with defecation at baseline to 4 d/week with defecation at week 4. The change from baseline in d/week with defecation was 1·54, 1·30 and 1·15 for the one billion, ten billion and placebo group, respectively (Fig. 4). For probiotic treatment overall, there was a statistically significant effect of probiotic treatment over the 4-week treatment period (*P*=0·0065), with the average defecation frequency being significantly higher compared with placebo at all weeks (all *P*<0·05). Additional data on average defecation frequency are included in the online Supplementary Table S2.

### Gastrointestinal well-being

There were no statistically significant differences for GI well-being (online Supplementary Fig. S2). Results for the subgroups with different baseline defecation frequency and sex/hormonal status were similar (data not shown).

### Other end points

Abdominal pain and bloating decreased during the study in all the groups. The average weekly sum score from baseline to week 4 decreased by 42–44 and 39–42 % for pain and bloating, respectively, but no difference between treatments was found (data not shown).

During the 4-week intervention period, the consistency of stools became softer in all the treatment groups with the median stool type increasing from 2·0 at baseline to 3·0 at week 4 in all the groups. Analysis of week 4 showed an overall treatment effect (*P*=0·046) and a trend for slightly softer stool in the one billion group (*P*=0·056).

### Adverse events

In total, 337 AE in 233 (18·7 %) subjects were recorded during the study. Of these, seventeen events in fourteen (1·1 %) subjects were assessed by the Investigator as related to the study treatment. The majority of the related events (sixteen events in thirteen subjects) were GI disorders, which were expected as one important inclusion criteria was abdominal discomfort. In total, three non-related AE were defined as serious. There were no obvious differences between the treatment groups in the number of AE or the number of subjects with events. Based on these data, the BB-12^®^ probiotic strain is considered safe. Details on related AE are included in the online Supplementary Table S3.

## Discussion

The results of this study are the outcome of a large randomised trial investigating the effects of the probiotic strain *Bifidobacterium animalis* subsp. *lactis*, BB-12^®^, in healthy subjects with a low defecation frequency and abdominal discomfort. The probiotic supplementation increased the probability of having a defecation frequency above baseline for at least 2 of the 4-week intervention with an overall OR of 1·31 (95 % CI 0·98, 1·75). In the PP population, statistically significant OR of 1·52 and 1·43 were found in the one billion group and for treatment overall, respectively. The probability of having an increase in defecation frequency of at least 1 d/week for at least 2 of the 4-week intervention was significantly increased with OR between 1·50 and 1·61 for both doses and treatment overall. Furthermore, a statistically significant effect on average defecation frequency was found with higher frequency at all weeks for probiotic treatment overall.

To our knowledge, the results presented in this study represent the largest currently available data set from a randomised controlled trial investigating the effect of probiotics on bowel habits and GI symptoms in healthy subjects. The observed effect of the probiotic strain BB-12^®^ on average defecation frequency confirms previously published results on this strain^(^ ^19^ ^–^ ^21^ ^)^.

We observed very high placebo response rates in this study, although similar placebo effects have previously been reported in both healthy subjects and IBS patients^(^ ^17^ ^,^ ^25^ ^,^ ^26^ ^,^ ^33^ ^)^. Initially, we defined a responder as a subject with an increase over baseline in defecation frequency; however, tightening the criteria for being a responder to an increase from baseline of at least 1 d/week reduced the placebo effect considerably. This could indicate that the initial responder definition, although considered relevant, may have been too easy to reach, resulting in the high placebo effect observed in all the analyses using this definition, and confirms the relevance of performing the *post hoc* analysis using the tightened responder definition.

Although the ITT analysis is essential because it is conservative, reflects clinical practice and increases generalisability, it introduces heterogeneity because non-compliant and compliant subjects are analysed together. The PP analysis is a useful supportive analysis to estimate the non-diluted treatment effect, and especially in nutritional studies where the expected effect sizes may be smaller than that for a medicinal product this is important. When the ITT and PP analyses come to essentially the same conclusions, as in the present study, confidence in the study results are generally high^(^ ^34^ ^)^.

A subgroup with baseline defecation frequency <3 d/week was defined *a priori*. The cut-off for a defecation frequency of <3 d/week was chosen based on the Rome III criteria for functional constipation^(^ ^7^ ^)^. Subjects with such a low defecation frequency may be more unlikely to respond to a probiotic and require specific treatment with laxatives^(^ ^35^ ^)^. However, although subjects with a defecation frequency of <3 d/week at first sight seemed not to benefit from the probiotic treatment in the present study, results from the *post hoc* analysis showed that when the criteria for being a responder was tightened the responder rates were more similar across subgroups and were of the same magnitude as for the whole population. An explanation may be that the placebo effect in the subgroup with the lowest baseline defecation frequency was initially almost 90 % and was reduced considerably after tightening the criteria, which may have disguised the benefit of the probiotic treatment. Therefore, our data show that even subjects with a very low defecation frequency gained benefit from a daily dose of the probiotic strain BB-12^®^.

Taken together, the results from the ITT and PP analyses, the *post hoc* results and the analysis of average defecation frequency demonstrate a consistent and clinically relevant effect of the probiotic strain BB-12^®^.

No difference was observed between the two doses tested. However, it may not be appropriate to expect probiotics to exhibit the sort of dose–response effects seen with pharmacological agents designed to affect a single target site. Probiotics are living organisms that produce many different metabolites and interact with many different receptors, molecules and cell types, and therefore may display an atypical dose–response relationship, which can also be seen with other well-known compounds such as corticosteroids, morphine and vitamins. Although the role of probiotics in health and disease is a fast-moving research area, the underlying mechanisms are still poorly understood and need further investigation. Furthermore, only few studies testing probiotics for different GI indications have investigated dose–response relationships with mixed results^(^ ^36^ ^–^ ^42^ ^)^. Studies of probiotics for antibiotic-associated diarrhoea have shown increased effect with higher dose^(^ ^41^ ^,^ ^42^ ^)^, whereas a study of discomfort symptoms in IBS patients indicated a missing dose–response relationship of probiotics, as an effect was observed at a medium dose level and no effects were observed for a lower or a higher dose^(^ ^38^ ^)^. The results from the present large study suggest that a ceiling effect exists for defecation frequency and has been reached with the one billion dose of the probiotic strain BB-12^®^.

The interim analyses resulted in a recommendation to stop first the one billion arm and next the ten billion arm. These analyses were based on the initial responder definition that did not show the expected difference in responder rates. Furthermore, as the responder rates in the two dosage arms proved to be similar, closing the one and ten billion arms at different interim analyses merely indicates that the test statistics have been close to the boundary values and not that the doses perform differently.

In spite of the interim results, we consider the results of this study important. Our study was powered to detect a 10 % difference in responder rates, which was the exact difference found when tightening the criteria for being a responder. Furthermore, when studying chronic conditions, regression towards the mean is generally a challenge^(^ ^30^ ^)^ and might have led to an underestimation of the true effect of the probiotic strain. Although the overall, global prevalence of IBS and functional constipation is 10–15 %, many more people have undiagnosed functional GI disorders as they do not seek medical care and a large number of healthy people have similar mild abdominal symptoms^(^ ^8^ ^,^ ^9^ ^)^. Therefore, supplementation of the probiotic strain BB-12^®^ may provide an easy, accessible and safe remedy that can benefit a large population for whom no effective alternatives exist^(^ ^43^ ^–^ ^45^ ^)^.

Other probiotic studies in healthy subjects with minor digestive symptoms have mainly focused on GI well-being and symptoms with mixed results^(^ ^25^ ^,^ ^26^ ^,^ ^39^ ^)^. In the present study, we did not see an effect on GI well-being, which may be partly explained by the use of global assessment of subjects’ relief of abdominal discomfort. In the past few years, it has been debated whether this outcome is the best way to evaluate treatment effect^(^ ^15^ ^,^ ^46^ ^,^ ^47^ ^)^, and consequently recently published guidelines no longer recommend the global rating as the primary end point in treatment trials for IBS^(^ ^31^ ^,^ ^32^ ^)^. This may also be evident for a study population with minor GI symptoms.

At present, neither the aetiology nor the pathophysiology of functional GI disorders is clear, and many factors such as genetic, immune, inflammatory, neurological and psychological may play an important role, although none of these are yet completely understood^(^ ^48^ ^,^ ^49^ ^)^. As many pathogenic factors contribute in different combinations, it is unlikely that one drug, acting on one pathophysiological mechanism, will be able to treat all the symptoms of IBS. Rather, different products or products acting on multiple pathophysiological mechanisms may be needed for targeting the different pathophysiological mechanisms behind various symptoms^(^ ^45^ ^,^ ^48^ ^)^. This may also explain the results of the present trial, where the probiotic product had an effect on defecation frequency, whereas no effect was observed on GI well-being. Unfortunately, there is currently no effective way to identify different pathophysiological subgroups in order to select a subpopulation with a higher likelihood of response to a specific intervention. Conducting clinical studies in IBS patients or populations with minor GI symptoms as in the present study is not only challenged by an underlying heterogeneity but also by fluctuations in symptom presentation, which tends to regress towards the mean during the course of a clinical study. In addition, efficacy measures still rely on patient-rated outcomes^(^ ^6^ ^,^ ^30^ ^,^ ^32^ ^)^. The mechanisms involved in the effect of probiotics on GI functions need further investigation in parallel with the elucidation of the underlying pathophysiology of functional GI disorders.

In conclusion, the results of this study strongly support a clinically relevant benefit of the probiotic strain *Bifidobacterium animalis* subsp. *lactis*, BB-12^®^, on defecation frequency in healthy subjects with low defecation frequency and abdominal discomfort. The efficacy of the two doses was similar, indicating that there is a ceiling effect – the reason for this being unknown at this point in time. More research is needed to elucidate this further and to understand how to best assess GI well-being in future studies.

## Supplementary material

For supplementary material accompanying this paper visit http://dx.doi.org/10.1017/S0007114515003347. click here to view supplementary material
