# Supplementary material for: Effect of the probiotic strain Bifidobacterium animalis subsp. lactis, BB-12®, on defecation frequency in healthy subjects with low defecation frequency and abdominal discomfort: a randomised, double-blind, placebo-controlled, parallel-group trial
Source: Br J Nutr. 2015 Sep 18;114(10):1638–46. doi: 10.1017/S0007114515003347 (PMC4657032; doi:10.1017/S0007114515003347)
Supplement: Supplementary file 1 [file S0007114515003347sup001.docx]

Table S1 Inclusion and exclusion criteria

| Inclusion criteria |
| --- |
| 1. Healthy men or women |
| 1. ≥ 18 - 70 years |
| 1. BMI between 19 – 35 kg/m^2^ (both inclusive) |
| 1. General abdominal discomfort/complaints weekly during the past month |
| 1. Stool frequency on average between 2-4 days per week with any number of defecations on these days |
| 1. Subject registered with national health insurance or benefiting from another similar system |
| 1. Subject registered in the national file of volunteers participating in biomedical research, if applicable |
| Subjects were eligible for randomization if they fulfilled the following two criteria based on the run-in diary: |
| 1. Stool frequency: had to be on average between 2-4 days per week (or 4-8 days for the 14-days run-in phase) with any number of defecations on these days |
| AND |
| 1. Gastrointestinal discomfort symptoms: had to be a composite symptom score of 5.0 or more per day on average on a 5-point Likert scale OR there had to be 5 (of 14) days where at least one of the symptoms had to be of severe (grade 3) intensity (symptoms marked as severe may differ from day to day). |
| Exclusion criteria |
| 1. History of hypersensitivity to any of the ingredients of the study products or lactose intolerance |
| 1. History or diagnosis of GI disease (e.g. gastric or duodenal ulcers, irritable bowel disease, colon cancer) or irritable bowel syndrome (IBS) or history of complicated GI surgery that might have an effect on gastrointestinal tract function |
| 1. Depressive disorder or hypochondria |
| 1. Any physical abnormality or medical condition that might have an effect on GI discomfort or on the evaluation of effects of the consumption of the investigational products |
| 1. Participation in any other clinical study within 1 month prior to enrolment in this study and until 2 weeks after the study |
| 1. Oral antibiotics within 4 weeks prior to the screening visit |
| 1. Use of any drugs, including over the counter products, for digestive symptoms such as anti-spasmodics, laxatives, anti-diarrheic drugs within 2 weeks prior screening, unless (as per Investigator’s discretion) in stable dose 4 weeks prior to screening |
| 1. Use of food or herbal supplements for digestive symptoms or large doses of vitamins and minerals unless in stable dose 4 weeks prior to screening |
| 1. Change of dietary habits within 4 weeks prior to the screening visit, e.g. start of fibre-enriched diet |
| 1. Not willing or able to provide written informed consent for participation in the study after being informed by the study personnel about the aim, course and possible risks of the study |
| 1. Not willing to give consent for transmission of personal "pseudonymised" data |
| 1. For women: Not willing and able to use a reliable contraceptive method. Reliable methods for women are hormonal contraceptives (oral or implants), surgical intervention (e.g. tubal ligation), intrauterine device (IUD), condoms and sexual abstinence. Women must use reliable methods from the screening examination until after the final examination |
| 1. For women: Pregnancy, lactation or wish to become pregnant |
| 1. Subjects who are unable to comply with the requirements of the study or who in the opinion of the study personnel should not participate in the study |
| 1. Subject having received more than 4500 Euros as indemnity for participation in clinical studies in the last 12 months, including participation in the present study (relevant only for France) |

**Figure S1** Responders in defecation frequency in subgroups of gender/hormonal status (ITT analysis)

n, number of subjects; OR, odds ratio for being a responder; CI, confidence interval; ITT, intention-to-treat; PP, per-protocol; CFU, Colony Forming Units. * Number of subjects (% responders)

A responder was defined as a subject with a weekly stool frequency above baseline / ≥1 day per week above baseline for at least 50% of the time, i.e. for at least 2 of the 4 weeks treatment period. Due to missing data 6 subjects (0.5%) could not be classified as responders or non-responders.

**Table S2** Average defecation frequency in days/week with defecation (ITT analysis)

|  | 1 billion CFU  n=343 | | 10 billion CFU  n=452 | | Probiotics overall  n=795 | | Placebo  n=453 | |
| --- | --- | --- | --- | --- | --- | --- | --- | --- |
|  | Mean | SD | Mean | SD | Mean | SD | Mean | SD |
| Baseline | 2.9 | 0.6 | 2.9 | 0.6 | 2.9 | 0.6 | 2.9 | 0.6 |
|  |  |  |  |  |  |  |  |  |
| Week 1 | 3.5 | 1.3 | 3.5 | 1.3 | 3.5 | 1.3 | 3.3 | 1.2 |
| p-value* | 0.19 | | 0.033 | | 0.039 | |  |  |
|  |  |  |  |  |  |  |  |  |
| Week 2 | 4.2 | 1.4 | 4.1 | 1.4 | 4.1 | 1.4 | 3.8 | 1.4 |
| p-value* | 0.0004 | | 0.0037 | | 0.0002 | |  |  |
|  |  |  |  |  |  |  |  |  |
| Week 3 | 4.3 | 1.5 | 4.2 | 1.5 | 4.2 | 1.5 | 4.0 | 1.5 |
| p-value* | 0.0080 | | 0.053 | | 0.0086 | |  |  |
|  |  |  |  |  |  |  |  |  |
| Week 4 | 4.4 | 1.5 | 4.2 | 1.5 | 4.3 | 1.6 | 4.0 | 1.5 |
| p-value* | 0.0002 | | 0.087 | | 0.0021 | |  |  |

ITT, intention-to-treat; CFU, colony forming units; n, number of subjects; SD, standard deviation

*p-values from repeated GEE analysis with Poisson distribution.

**Table S3 Average stool consistency and number of straining episodes (ITT analysis)**

|  | 1 billion CFU  n=343 | | 10 billion CFU  n=452 | | Placebo  n=453 | |
| --- | --- | --- | --- | --- | --- | --- |
|  | Mean | SD | Mean | SD | Mean | SD |
| **Stool consistency**  **(Bristol Stool Form Score)** |  |  |  |  |  |  |
| Baseline | 2.38 | 1.10 | 2.34 | 1.08 | 2.28 | 0.99 |
| Week 4 | 3.32 | 1.23 | 3.11 | 1.20 | 3.15 | 1.19 |
| p-value^1^ | 0.056 | | 0.61 | |  | |
|  |  |  |  |  |  |  |
| **Number of straining epiodes (times/week)** |  |  |  |  |  |  |
| Baseline | 2.49 | 1.04 | 2.45 | 1.01 | 2.58 | 1.04 |
| Week 4 | 2.08 | 1.83 | 2.06 | 1.87 | 1.99 | 1.85 |
| p-value^2^ | 0.31 | | 0.25 | |  | |

ITT, intention-to-treat; CFU, colony forming units; n, number of subjects; SD, standard deviation

^1^ p-value from analysis of variance on ranked data at Week 4.

^2^ p-value from repeated GEE with Poisson distribution.

**Figure S2** Responders in GI well-being (ITT and PP analysis)

n, number of subjects; OR, odds ratio for being a responder; CI, confidence interval; CFU, Colony Forming Units. * Number of subjects (% responders)

A responder was defined as a subject with relief (somewhat relieved or markedly relieved) for at least 50% of the time, i.e. for at least 2 of the 4 weeks treatment period. Due to missing data 13 subjects (1.0%) could not be classified as responders or non-responders.

**Table S4** Related adverse events reported

| Treatment  System Organ Class | Preferred term | 1 billion CFU  n=343 | 10 billion CFU  n=452 | Placebo  n=453 |
| --- | --- | --- | --- | --- |
|  |  | N (%) E | N (%) E | N (%) E |
| All Adverse Events |  | 6 (1.7) 7 | 7 (1.5) 8 | 1 (<1) 2 |
|  |  |  |  |  |
| Gastrointestinal Disorders | Abdominal discomfort | 0 (0) 0 | 0 (0) 0 | 1 (<1) 1 |
|  | Abdominal pain | 3 (<1) 3 | 1 (<1) 1 | 0 (0) 0 |
|  | Abdominal pain upper | 1 (<1) 1 | 1 (<1) 1 | 0 (0) 0 |
|  | Diarrhea | 1 (<1) 1 | 2 (<1) 2 | 0 (0) 0 |
|  | Dyspepsia | 0 (0) 0 | 1 (<1) 1 | 0 (0) 0 |
|  | Flatulence | 0 (0) 0 | 1 (<1) 1 | 0 (0) 0 |
|  | Nausea | 2 (<1) 2 | 1 (<1) 1 | 1 (<1) 1 |
| Skin and Subcutaneous Tissue Disorders | Acne | 0 (0) 0 | 1 (<1) 1 | 0 (0) 0 |

CFU, colony forming units; n, number of subjects; N, number of subjects in the treatment group having the event; %, percentage of subjects in treatment group having the event, E, number of events, events could be counted only in one group.

Relatedness was assessed by the Investigator. Related = events with causality rated as certain, probable or possible. No events were rated as certain.

System organ class was coded in MedDRA version 15.
